# The urge to publish more and its consequences

**DOI:** 10.1186/2008-2231-22-53

**Published:** 2014-06-30

**Authors:** Mohammad Abdollahi, Armen Yuri Gasparyan, Soodabeh Saeidnia

**Affiliations:** 1 Faculty of Pharmacy and Pharmaceutical Sciences Research Center, Tehran University of Medical Sciences, Tehran 1417614411, Iran; 2 Council Member of the European Association of Science Editors, London, UK; 3 Departments of Rheumatology and Research & Development, Dudley Group NHS Foundation Trust, Teaching Trust of University of Birmingham, UK, Russells Hall Hospital, Dudley, West Midlands DY1 2HQ, United Kingdom; 4 Medicinal Plants Research Center, Tehran University of Medical Sciences, Tehran 1417614411, Iran

## 

In the era of “big science”, all researchers, academics and students are under pressure to publish more and to report more research activities for successful grant applications, academic promotion, and course graduation. To meet the ever-increasing publication activities, thousands of new publishers have sprung up globally, and the number of online and subscription journals has increased exponentially. At the same time, temptation to publish more at all costs has led to epidemics of unethical conduct and inevitable retractions, which question the validity of current evidence-base [1]. Reasons for retractions include, but not limited to fraud, plagiarism, multiple submissions and duplicate publications, violation of copyrights and ethical norms of research, and inappropriate authorship [2]. Uncovered cases of misconduct and violation of publication ethics are increasing at rapid pace due to the digitization and open access movement in the last two decades [1]. And it is expected that the landscape of publications and their indexing and citation records in bibliographic databases will change substantially in the coming years.

Large amount of funding for research, publishing and archiving activities comes from pharmaceutical agencies, supporting individuals and their research and academic institutions. Too often, pharmaceutical agencies hire medical writers and professional experts for writing research reports and guidelines on drugs and medical technologies produced or promoted by the agencies. Informing readership about relationships between authors and pharmaceutical agencies is an ethical obligation, which should be regarded for the sake of transparency and safety of patients [3,4]. Non-disclosure of relevant relationships with pharma is a misconduct with far-reaching healthcare consequences that cannot be avoided even after corrections or retractions of unethical publications. The authors have to explicitly disclose any conflict at the manuscript submission by filling the structured form provided by the International Committee of Medical Journal Editors [5].

Global editorial associations keep a close eye on research and publication misconduct. For example, the Committee on Publication Ethics (COPE) has a collection of appalling cases of misconduct, which may help avoid similar cases by improving awareness among young and seasoned researchers, authors, reviewers, and editors [6]. Responsibility for accurate and unbiased research reporting lies not only with authors, but also with all other stakeholders of scientific communications. Research and academic institutions are obliged to educate their authors and to inform about publishing ethics and consequences of biased and fraudulent publications. Reviewers and science editors, in turn, have to carefully evaluate correctness of research data and transparency of authorship, contributorship, and disclosures of ethical approvals, funding, and conflicts of interests. Ethical concerns may arise at any point throughout the manuscript processing or post-publication, and if so, responsible evaluators have to consult relevant guidelines of COPE and act accordingly [7]. Each case of misconduct should be discussed in a collegiate way with all stakeholders of research publications to avoid similar cases in the future.

Research misconduct may take a variety of forms, which can be minor or major. The U.S. National Science Foundation distinguished the following types of research misconduct: fabrication (reporting made-up results), falsification (manipulation of the research materials, equipment, or processes), and plagiarism (utilizing someone else’s text or ideas without appropriate crediting) [8,9]. Perhaps the most important point here is the intention, which leads to the misconduct and unethical publication (Figure 1). Intentionally misleading readership by plagiarizing large parts of previous publications, presenting fabricated data, or omitting key negative data are matters of gross misconduct, which require proper actions by reviewers, editors, and publishers. Misinterpretation of statistical data and omission of data on adverse effects of drugs are also unethical acts, misleading non-expert readership [10]. Minor forms of misconduct are text recycling due to poor language skills or “academic laziness”, which often stem from the urge to publish more, and can be prevented by sharpening language skills and properly organizing the writing work. Whether or not such a “minor” misconduct requires manuscript withdrawal or retraction depends on the intention and several other factors. In any case, relying on available guidance from COPE may help reach an ethically acceptable decision [11,12].

**Figure 1:**
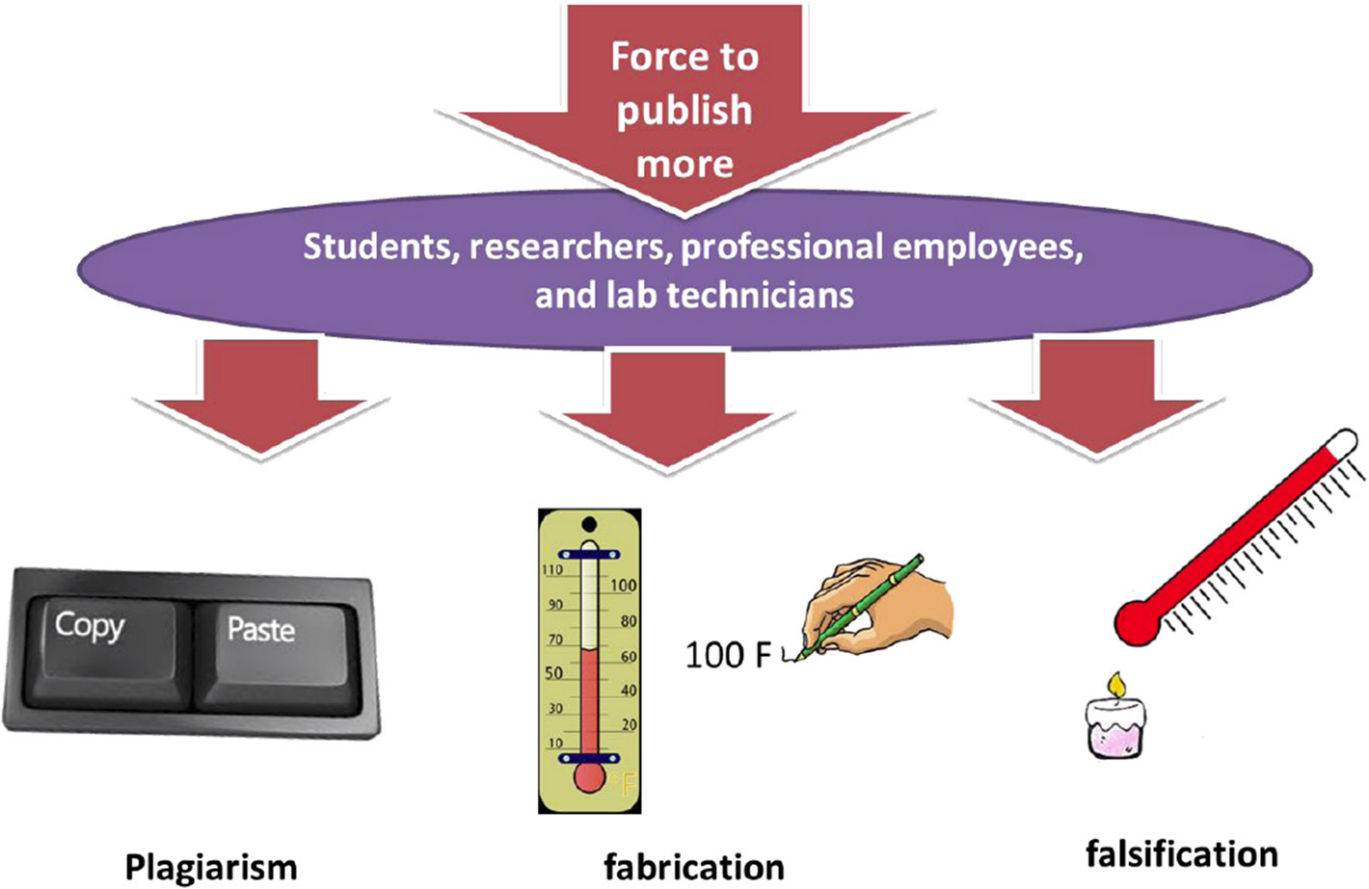
Types of research misconduct by the U.S. National Science Foundation, which may take place as a consequence of the urge to publish more.

Current “obsession” with impact factors and individual impact indicators has led to the urge to produce more “groundbreaking” research data and target high-ranking scholarly journals. Authors, who publish in high-impact periodicals, are likely to attract more citations and secure more funding from grant holders. The wealthiest research grant providers prioritize widely-visible and most-impacting publications, thus forcing researchers and their institutions to publish more in top journals (Figure 1). As a result, top-ranking journals such as *Science, Nature, Cell,* and others primarily suffer from fraudulent and unethical publications, eventually subjected to retractions [1].

At the other extreme, authors, who wish to build up their academic profile and publish at all costs, may be tempted to circumvent the mainstream journals with tough peer review and to submit their manuscripts to the so-called predatory journals with no quality control or soft, decorative peer review (Figure 2). The main aim of such journals is to sell a space for substandard research and poorly edited papers and to attract inexperienced authors [13]. Predatory publishers may also claim that they publish journals that have certain impact indicators, often calculated by newly launched bogus impact agencies. Research and academic institutions should take an active stance against such journals, and familiarize their authors with what constitutes a quality and influential journal and endorse widely acceptable traditional and alternative impact indicators [14]. One such impact indicator is the *h*-index, which is universally applicable for evaluating publication activity and citability of individuals, academic departments, institutions, and countries. Its bi-dimensional origin is well fitted with the concept of publishing citable papers useful for the scientific community.

**Figure 2:**
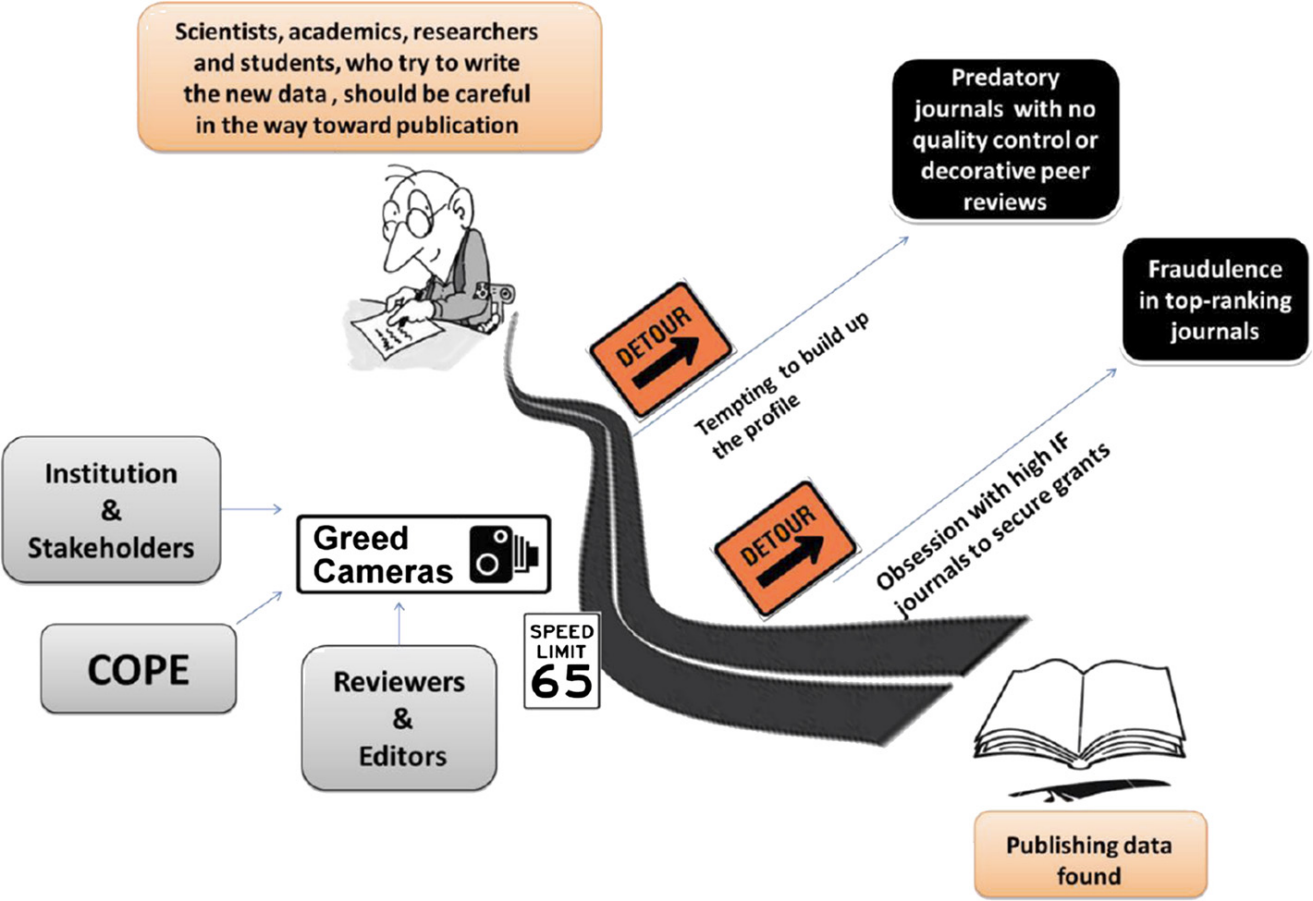
Consequences of the urge to publish more and the role of evaluators.

Authors should be aware of functional characteristics of online search platforms, bibliographic databases, and archiving hubs. Open-access publishing provides opportunities for rapid dissemination of scholarly information. However, not all online journals are indexed in evidence-based databases and are capable of properly archiving papers. Biomedical authors, who wish to address readership, interested in reading and citing their papers, should target MEDLINE-indexed journals in the first place. Publishing in journals indexed in MEDLINE and simultaneously archived in PubMed Central is an added value as the latter is an ideal platform for open-access publishing models and permanent conservation of scholarly information. Papers archived in PubMed Central can be retrieved from PubMed and Web of Knowledge search platforms. However, journal visibility in these platforms differs from indexing in MEDLINE and Web of Science bibliographic databases. Publishers should explicitly inform potential authors about correct indexing and archiving status of their journals and avoid manipulating and charging for non-indexed publications.

Exploring options for disseminating scientific ideas in an ethical and well-informed academic environment is a big issue for both researchers and publishers [15]. Obviously, educating all stakeholders of scientific communications about effective research reporting, open archiving and re-using published sources, indexing journals, and calculating impact indicators is the logical way out of unethical and flawed publishing environment. Priority of well-checked and edited publications in journals indexed in relevant databases and web platforms should become a guiding point for all those who rush to publish more.

## Competing interests

Mohammad Abdollahi (MA) is the Editor-in-Chief of DARU and chose the topic to be written based on his editorial experiences. MA is a Trustee Council Member of Committee on Publication Ethics (London, UK) and the views expressed in this publication do not necessarily reflect the views of the COPE. Armen Yuri Gasparyan is the Chief Editor of European Science Editing and a Council Member of the European Association of Science Editors (London, UK) and the views expressed in this publication do not necessarily reflect the views of the EASE.

## Authors’ contributions

All authors contributed equally. All authors read and approved the final manuscript.
